# Minimizing Delamination in CFRP Laminates: Experimental and Numerical Insights into Drilling and Punching Effects

**DOI:** 10.3390/polym17223056

**Published:** 2025-11-18

**Authors:** Murat Demiral, Tamer Saracyakupoglu, Burhan Şahin, Uğur Köklü

**Affiliations:** 1 College of Engineering and Technology, American University of the Middle East, Egaila 54200, Kuwait; 2 Turkish Aerospace Industries, Ankara 06980, Türkiye; dr.tamer@tamersaracyakupoglu.com.tr (T.S.); burhan-03@hotmail.com (B.Ş.); 3 Faculty of Engineering, Department of Mechanical Engineering, Karamanoglu Mehmetbey University, Karaman 70100, Türkiye; ugurkoklu@kmu.edu.tr; 4 Faculty of Engineering and Architecture, Department of Mechanical Engineering, Recep Tayyip Erdogan University, Rize 53100, Türkiye

**Keywords:** CFRP laminates, delamination, drilling and punching, continuum damage mechanics, cohesive zone modeling

## Abstract

Carbon fiber-reinforced polymer (CFRP) laminates are extensively utilized in aerospace and advanced engineering fields because of their outstanding strength-to-weight ratio and superior fatigue durability. However, despite their high in-plane strength and stiffness, CFRP laminates are inherently susceptible to delamination. This weakness stems from the relatively low interlaminar strength of the resin-rich interfaces between layers compared to the much stronger in-plane fiber reinforcement. During mechanical processes such as drilling and punching, out-of-plane stresses and interlaminar shear forces develop, concentrating at these weak interfaces. This study investigates the delamination behavior of CFRP laminates with 3 to 7 plies under drilling and punching, focusing on the effects of ply count and drilling speed. Experimental tests were conducted using an 8 mm punch and drill bit at 2500, 3000, and 3500 rpm, reflecting typical workshop practices for M8 fastener holes. Scanning electron microscopy (SEM) analyses at different magnifications were used to quantify delamination extent. A three-dimensional finite element model was created in ABAQUS/Explicit, integrating the Hashin failure criterion to predict damage initiation within the plies and cohesive surfaces to simulate interlaminar delamination. The analyses show that with proper support, punching can approach the damage levels of drilling for thin CFRP plates, but drilling remains preferable for thicker laminates due to better integrity and tool longevity.

## 1. Introduction and Motivation

Carbon fiber reinforced-polymer (CFRP) composites have become indispensable materials in a wide range of high-performance industries due to their exceptional mechanical properties, including high strength-to-weight ratios, stiffness, and corrosion resistance. These properties make CFRP composites highly desirable for aerospace, automotive, and structural applications, where minimizing weight while maintaining high performance is crucial. However, the superior in-service behavior of CFRP comes with significant challenges in secondary manufacturing processes, especially in hole-making operations such as drilling and punching, which are critical for assembly using mechanical fasteners. The anisotropic and heterogeneous nature of CFRPs, resulting from the combination of carbon fibers and polymer matrices, introduces complex interactions during machining that can lead to defects such as delamination, fiber pull-out, and matrix cracking [1].

Previous studies have explored various aspects of CFRP machining, providing essential insights into how cutting parameters and tool geometry affect damage evolution and tool wear. For instance, Goutham et al. [2] systematically investigated the effects of drilling parameters and tool geometry on delamination and tool wear when machining carbon fiber-reinforced epoxy composites. Their two-stage experimental approach, involving a full factorial design followed by tool performance comparisons, revealed that uncoated tungsten carbide drills with a 90° point angle and 35° helix angle consistently produced lower delamination and exhibited minimal wear. Notably, coated tools underperformed due to premature coating erosion, while a combination of low feed rates and high cutting speeds minimized thrust force and drilling-induced damage. Fernandes Perez et al. [3] extended this understanding by analyzing the relationship between cutting parameters, tool wear mechanisms, and drilling-induced damage in CFRP composites drilled with diamond-coated carbide drills. They observed that coating detachment and subsequent abrasive wear of the substrate directly affected delamination and thrust force, with higher cutting speeds promoting more efficient hole production but also accelerating tool wear. Their predictive models for thrust force and delamination provide valuable tools for optimizing cutting strategies to enhance tool life and machining efficiency.

The complexity of CFRP machining, arising from its anisotropic behavior and thermomechanical interactions, has driven the development of theoretical and numerical models to predict machining responses. Song et al. [4] provided a comprehensive review of cutting force models for CFRP composites, categorizing them into macro-mechanical, mechanistic, micro-mechanical, and numerical approaches. While mechanistic models remain popular for their adaptability and practical utility, they often struggle to capture complex interactions such as tool wear, heat generation, and multi-axial stresses, which can significantly influence delamination and surface integrity. Numerical models, particularly those using finite element methods (FEM), are increasingly recognized for their predictive capabilities and ability to incorporate detailed material behavior, albeit at the cost of increased computational resources.

Zadafiya et al. [5] explored challenges and critical parameters in CFRP drilling, emphasizing the roles of drill geometry, feed rate, tool coatings, and cooling strategies in affecting thrust force, surface quality, and hole defects. Their work highlighted the effectiveness of cryogenic cooling and advanced drill geometries in reducing machining damage, while stressing the importance of delamination factor metrics in evaluating hole quality. Jia et al. [6] contributed to this understanding by developing a thermomechanical analytical model that integrates thermal effects into the prediction of critical thrust force during drilling of unidirectional CFRP laminates. Their model accounts for the composite’s anisotropy, tool geometry, and thermal interactions, validated through simulations and experiments, showing that delamination risk increases with rising drilling temperatures and that critical feed rates decrease with fewer uncut plies or larger drill diameters.

Non-destructive evaluation methods for assessing drilling-induced delamination were investigated by Maleki et al. [7], who compared three approaches in natural fiber composites and identified image processing with a flatbed scanner as the most accessible, cost-effective, and accurate technique for identifying the size and shape of delamination. Although focused on natural fibers, their method offers a practical approach for rapid delamination assessment in CFRPs as well. Can et al. [8] examined the dynamic behavior of screwed joints in CFRP laminates under low-velocity impact loading, revealing shifts in failure modes from interlaminar delamination to push-out delamination as screw pitch increased. Their findings underscored that CFRP screwed joints can match or even exceed the performance of riveted or bonded joints, provided that high-quality holes are achieved.

The literature also highlights significant advances in numerical modeling of composite plates under drilling conditions [9,10,11,12,13,14]. A detailed three-dimensional finite element study was conducted by Phadnis et al. [13], who modeled the progressive damage in CFRP laminates during drilling using an explicit finite element framework with damage mechanics criteria. Their approach incorporated cohesive zone elements to capture delamination, providing accurate predictions of cutting forces and damage evolution. Isbilir et al. [14] created a 3D mesoscale finite element model to simulate drilling in carbon fiber-reinforced laminates, focusing on the impact of cutting speed and feed rate on thrust force, torque, and delamination. The model incorporated layered ply orientations and cohesive interfaces, and its predictions were validated through experimental drilling and digital image-based delamination analysis. Results indicated that increasing feed rate elevated all damage metrics, while higher cutting speeds reduced them. Although the model slightly underestimated thrust force and overestimated delamination, it proved effective for simulating composite drilling and emphasized the need for future thermo-mechanical modeling and more realistic friction modeling.

In contrast to drilling, the punching characteristics of composite plates were relatively less studied. Alnemrawi et al. [15] investigated the punching shear behavior of CFRP-strengthened reinforced concrete slabs, demonstrating that CFRP reinforcement improves ductility, load capacity, and crack confinement even under adverse thermal and structural conditions, emphasizing the importance of optimized hole quality and placement. The punching behavior of pure CFRP laminates made with thin-ply (62 g/m^2^) and standard (149 g/m^2^) UD prepregs was examined in the study of Ho and Yanagimoto [16]. Regardless of laminate thickness or punch speed, it was discovered that laminates created using normal prepregs needed a stronger punch force than thin-ply laminates. A useful metric for connecting punch performance to the laminates’ tensile and yield strengths was found to be punching resistance. Microscopic examination revealed that both laminate types had typical shear cracks and delamination, as well as shear-dominated failure processes. The findings provide credence to the viability of punching as a dependable and measurable technique for producing useful holes in pure CFRP laminates.

In a related study, Ayten et al. [17] investigated the effect of polyamide-6 (PA6) nanofiber interlayers on the quasi-static punching behavior of carbon fiber-reinforced epoxy composites. Using the vacuum-assisted resin transfer molding (VARTM) process, they found that the inclusion of PA6 nanofibers significantly improved the load-bearing capacity and energy absorption during punch shear tests. The nanofiber interlayers effectively restricted delamination and delayed failure initiation, demonstrating their beneficial role in enhancing the punching resistance and overall structural integrity of CFRP laminates.

More recently, Matsuda et al. [18] investigated the piercing damage in CFRP cross-ply laminates subjected to punch shear machining via impact loading, providing further insight into the underlying damage mechanisms during high-speed hole formation. Their study revealed that impact-induced punching generates complex through-thickness damage comprising matrix cracking, interlaminar delamination, and fiber breakage near the punch entrance and exit regions. The experimental observations showed that the extent of damage strongly depends on laminate stacking sequence, punch geometry, and impact energy. Furthermore, post-impact microscopic evaluations confirmed that the dominant failure mode transitions from interlaminar separation to fiber fracture as the impact energy increases. The results of Matsuda et al. [18] contribute valuable understanding of the dynamic punching behavior of CFRP laminates and emphasize the necessity of considering impact-induced damage when evaluating the integrity and residual performance of pierced composite structures.

Although drilling is the most commonly studied and used method for making holes in CFRP composites, the punching process has received far less attention in the literature. In fact, there is a noticeable gap when it comes to directly comparing the two techniques. Given the different ways each method interacts with the material—particularly in terms of deformation and damage—it is worth asking whether punching could offer a viable alternative to drilling. Exploring the performance of punching more thoroughly could help identify the situations where it might not only match but even outperform drilling, or at least serve as a complementary option. Such a comparison would provide valuable insights for expanding manufacturing options in composite applications. On the other hand, while numerous studies have addressed various aspects of hole-making in CFRP composites, there is a clear lack of systematic investigation into how ply count and drilling speed influence drilling performance and damage characteristics.

To address this gap, we conducted a combined experimental and numerical investigation. Our experimental work focused on comparing delamination behavior in 3- to 7-ply CFRP twill laminates subjected to punching and drilling operations using an 8 mm punch and drill bit, with drilling performed at 2500, 3000, and 3500 rpm. These speeds were chosen as they represent the most frequently used drilling speeds for CFRP in typical workshop settings. The 8 mm hole diameter corresponds to the standard size for M8 fasteners, which are widely used in aerospace applications due to their high load-bearing capacity and ease of assembly. After drilling and punching operations, we performed detailed scanning electron microscopy (SEM) analysis at magnifications of 100×, 1000×, and 5000× to evaluate the extent and nature of delamination. In parallel, we developed a comprehensive three-dimensional finite element model of the drilling and punching processes using ABAQUS 2021/Explicit. The model incorporated a Hashin damage initiation criterion combined with its complementary damage progression law. To accurately simulate interlaminar delamination, cohesive surfaces were applied at the interfaces between composite layers based on traction–separation behavior. This integrated modeling framework enabled us to reproduce the complex material behavior observed experimentally, providing valuable insights into the interactions between ply count, drilling speed, and damage evolution under different hole-making methods.

The structure of this paper is organized as follows. Section 2 details the experimental procedures, including the comparative SEM analysis of delamination behavior under different ply counts and drilling speeds. Section 3 outlines the theoretical foundation of the Hashin failure criterion and cohesive zone modeling, along with the development of the finite element model using ABAQUS/Explicit. Section 4 presents the experimental observations and numerical analysis. Finally, Section 5 provides the conclusions drawn from this integrated experimental and numerical investigation.

## 2. Experimental Study

**Figure 1:**
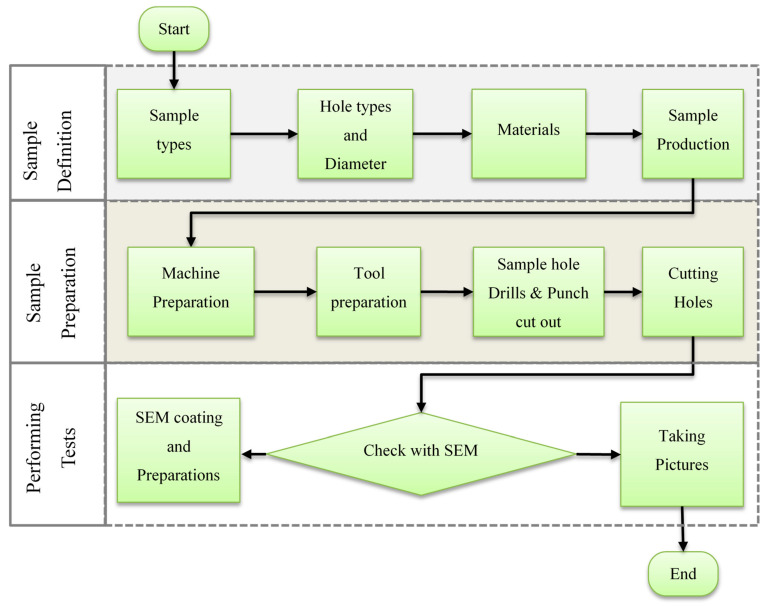
Methodical schema of experimental study.

The experimental studies are organized into three key sections, each crucial to the overall research process, as depicted in Figure 1. The first section is dedicated to the meticulous design of the test samples, where specific parameters and variables are established to ensure the validity and reliability of the experiments. In the second section, the focus shifts to the careful preparation of these samples for production, involving processes that ensure they meet the necessary quality standards and specifications for testing. The final section encompasses the application of various tests, followed by a thorough analysis of the results obtained, allowing for insightful interpretations and conclusions that contribute to the overall objectives of the study.

### Sample Design and Definition

The carbon fiber-reinforced polymer (CFRP) laminates for the samples have been designed at various angles and layers. The samples were woven in a twill pattern of 2 × 2 and produced using VTP H300 (SPM Prepreg Systems, Ankara, Türkiye) resin at different layers with angles of 90°, under a pressure of 6.9 KPa and a vacuum of 600 mm/hg at a temperature of 120 °C. The choice of VTP H300 prepreg is attributed to its low exothermic properties, which facilitate the production of thick parts, as well as its non-porous surface and strong adhesion characteristics, making it suitable for hot pressing. In this study, the most commonly used 4 to 7-layer ply carbon fiber composites were employed in air platforms.

**Figure 2:**
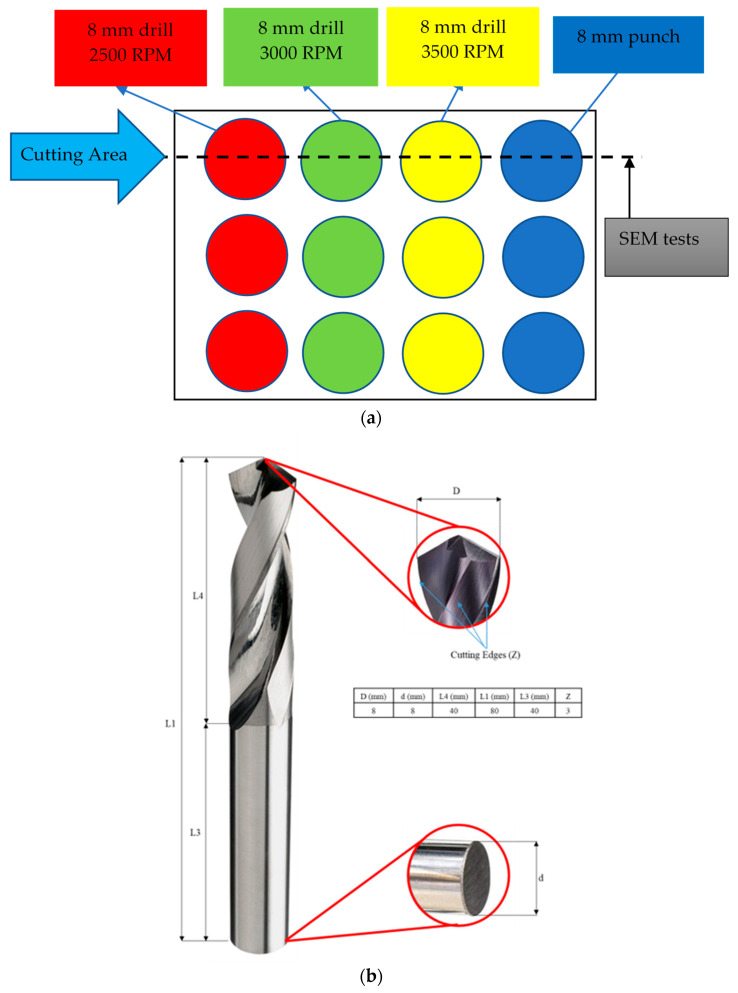
(**a**) Sample hole and RPM definitions, (**b**) drill bit specifications [19].

After the production of samples, it is aimed to create holes of the same diameter at different RPM speeds, as illustrated in Figure 2a, and subsequently conduct SEM tests by cutting these holes to observe the interior.

The drill bit is a Aerodrill ST3 (Cruing GmbH, Oberndorf, Germany) model with an 8 mm diameter, featuring a 3 PCD (Polly Crystal Diamond) 25° cutting edge, and conforms to the DIN 6535 shank form. It is manufactured from solid carbide material, as detailed in Figure 2b. In aviation, the common diameter used for rivets and holes is 8 mm.

**Figure 3:**
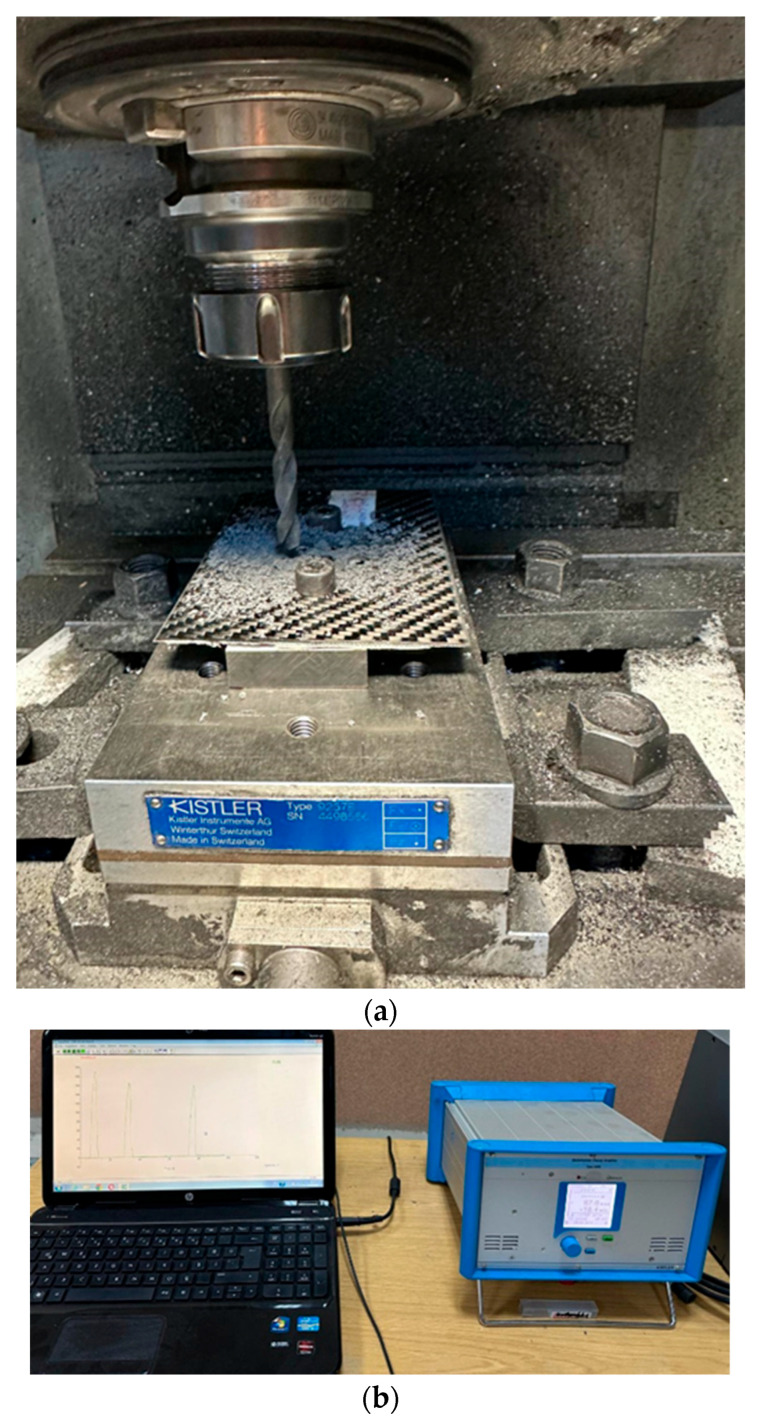
(**a**) The performed drilling process, (**b**) cutting force measurement.

Thrust force measurements during the drilling experiments (see Figure 3a) were conducted using the Kistler 9257B dynamometer, along with the 5070 amplifier and the 5697A data acquisition unit (see Figure 3b). Holes for SEM analysis from both processes were created using the Antares PX5 (C.M.S. S.p.A., Zogno, Italy) and Trumatic 2000R (TRUMPF (machine division), Ditzingen, Germany) machines. After hole formation, horizontal cutting was performed with a spiral tool. The samples were then examined using a Zeiss Evo 10 SEM (Nobil Metal S.p.A., Villafranca d’Asti, Italy). Before SEM imaging, a thin palladium coating was applied with the Sputter Coater SC7620 (Quorum Technologies Ltd., East Sussex, United Kingdom). Following coating, SEM images were captured at magnifications of 100×, 1000×, and 5000×. In total, 64 SEM images were analyzed, taken from four different holes across four samples at four distinct magnification levels. More representative SEM images have been added in Section 4 to better illustrate the observed damage patterns.

## 3. Numerical Modeling

**Figure 4:**
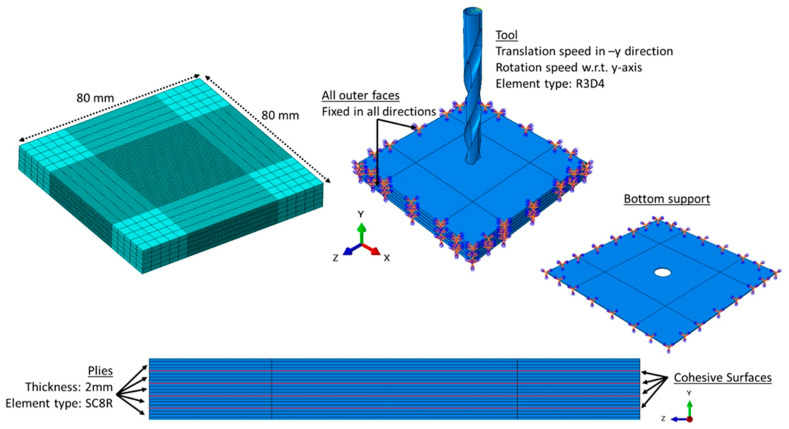
Three-dimensional finite element model for the drilling of the 5-ply CFRP plate.

This section outlines the numerical procedures adopted for the simulations. Drilling and punching operations on CFRP composite plates were simulated using the ABAQUS/Explicit finite element (FE) software [20]. Figure 4 illustrates the configuration of the developed FE model. A rectangular composite plate with dimensions of 80 mm × 80 mm × *t* (thickness) was modeled. To simulate interlaminar damage manifested as delamination between layers, cohesive interface elements were integrated into the model. The total thickness of a lamina, accounting for resin-rich regions and void content, was set at 2.0 mm. The composite laminas were discretized using 8-node linear hexahedral continuum solid elements (SC8R). To mitigate nonphysical zero-energy modes, enhanced hourglass control based on stiffness and distortion regulation was employed. Delamination and interface separation were captured using linear 3D cohesive surfaces. A fine mesh resolution of 0.5 mm × 0.5 mm × 0.5 mm was applied to the plies in the contact region near the punch or drill bit, while a coarser mesh was used in the remaining areas. To ensure numerical accuracy, a mesh convergence study was conducted using two additional mesh sizes: a finer mesh of 0.25 mm × 0.25 mm × 0.25 mm and a coarser mesh of 1.0 mm × 1.0 mm × 1.0 mm. The convergence was verified based on the force–displacement response, which showed less than a 5% variation compared with the results obtained using the finer mesh. The punch and drill tools as well as the bottom support were modeled as rigid bodies and meshed with 4-node bilinear rigid quadrilateral elements (R3D4). The drill bit and punch were modeled as a rigid body based on the assumption that their elastic stiffnesses (approximately 500–700 GPa) is considerably higher than that of the CFRP material used in this study (55 GPa). Consequently, the tool was idealized as non-deformable throughout the drilling process. This simplification not only reflects the negligible deformation of the tool relative to the workpiece but also substantially reduces the computational cost of the simulation—an approach widely adopted in similar numerical investigations.

Both tools were constrained to move only along the negative y-axis at a constant speed of 12.5 mm/s, with the drill additionally rotating about the y-axis at speeds ranging from 2500 to 3500 rpm. The rigid tools were discretized using a mesh size of 1.0 mm. The smallest element size—corresponding to the cohesive element thickness—governed the stable time increment. The backing plate was placed under the workpiece to support it.

For a composite plate composed of five layers, the full FE mesh comprised 195,684 elements, with the simulation requiring approximately 22.2 h on a high-performance computing (HPC) cluster utilizing 36 Intel Quad-core processors with 48 GB RAM each.

To simulate the interaction between the punch or drill bit and the composite plate, ABAQUS’s General Contact Algorithm was applied, employing a kinematic constraint enforcement approach to handle the normal contact behavior. Tangential interactions were governed by the Coulomb friction model, with a friction coefficient of *μ* = 0.4 [9]. All external surfaces of the composite laminate were constrained in every direction to prevent movement.

For damage modeling, a continuum damage mechanics approach was used to simulate intra-ply failure, while interlaminar failure was represented using the cohesive surfaces. The following sections provide detailed descriptions of both modeling techniques.

### 3.1. Intralaminar Damage

Fiber breakage and matrix cracking within the lamina were simulated using continuum damage mechanics with an orthotropic constitutive material model. To define damage initiation, a three-dimensional failure criterion derived from Hashin’s model [21] was implemented (Table 1).

Material point degradation begins when the equivalent stress reaches its maximum threshold, after which damage propagation is initiated. The model incorporates four distinct failure modes: fiber tension (*ft*), fiber compression (*fc*), matrix tension (*mt*), and matrix compression (*mc*). The governing equations for these modes are listed in Table 2, where directions 1 and 2 correspond to the in-plane fiber and matrix axes, respectively, and direction 3 denotes the out-of-plane axis. The failure indicators *f*_1_, *f*_2_, *f*_3_, *f*_4_ represent each mode, with failure occurring in a particular mode once its indicator attains a value of 1. The stress components are represented by $\sigma_{ij}$. The tensile and compressive strengths in the fiber direction are denoted by $X^{T}$ and $X^{C}$, respectively, while $Y^{T}$ and $Y^{C}$ indicate the corresponding strengths in the matrix direction. $S^{L}$ and $S^{T}$ refer to longitudinal and transverse shear strengths, respectively. The parameter α defines the influence of shear stress on the fiber tensile failure initiation criterion. It was assumed 1.0 in the simulations. Additional details of the model can be found in [22]. The material is assumed to behave elastically until one of the failure criteria is met.

Once a damage initiation criterion is met, continued loading leads to a reduction in the material’s stiffness matrix, a process referred to as damage evolution. In this study, damage evolution is modeled using a linear softening behavior. The progression of the damage variable for each failure mode *I* is defined by the following equation:

(1) $$d_{I}=\frac{\delta_{I,eq}^{f}(\delta_{I,eq}-\delta_{I,eq}^{0})}{\delta_{I,eq}(\delta_{I,eq}^{f}-\delta_{I,eq}^{0})}\;\;I\;=\;ft,\;fc,\;mt,\;mc$$

where $\delta_{I,eq}^{0}$ and $\delta_{I,eq}^{f}$ are the equivalent displacements at the initiation and completion of damage, respectively. $\delta_{I,eq}^{f}$ can be computed from $\delta_{I,eq}^{f}=2G_{I}/\sigma_{I,eq}$, where $G_{I}$ and $\sigma_{I,eq}$ are the fracture toughness and equivalent stress of the respective mode. The corresponding equivalent displacements and stresses for each failure mode are summarized in the table below.

The stresses within a damaged element are calculated using the classical stress–strain relationship, $\sigma =C_{d}\varepsilon$, where the stiffness matrix $C_{d}$ accounts for the stiffness degradation occurring during the damage evolution of the composite plies, defined as:

(2) $$C_{d}=\frac{\left[\begin{array}{ccc} E_{11}(1-d_{f}) & E_{11}\vartheta_{21}(1-d_{f})(1-d_{m}) & 0\\ E_{22}\vartheta_{12}(1-d_{f})(1-d_{m}) & E_{22}(1-d_{m}) & 0\\ 0 & 0 & G_{12}D(1-d_{s}) \end{array}\right]}{D}\;D\;=\;1-(1-d_{f})(1-d_{m})\vartheta_{12}\vartheta_{21}$$

where $E_{ij}$, $\vartheta_{ij}$, $G_{ij}$ denote the engineering constants of the material, while $d_{f}$, $d_{m}$ and $d_{s}$ are the damage variables representing the current states of fiber, matrix, and shear damage, respectively.

(3) $$d_{f}=\left\{\begin{array}{cc} d_{f}^{T} & if\;\sigma_{11}\geq 0\\ d_{f}^{C} & if\sigma_{11}<0 \end{array}\right.\;d_{m}=\left\{\begin{array}{cc} d_{m}^{T} & if\;\sigma_{22}\geq 0\\ d_{m}^{C} & if\sigma_{22}<0 \end{array}\right.\;d_{s}=1-\left(1-d_{f}\right)*(1-d_{m})$$

Table 3 shows the elastic, strength and damage values introduced in Abaqus for the simulation of composite plies.

The maximum in-plane characteristic length of an undeformed continuum element, defined as the square root of the product of its in-plane dimensions, must be smaller than the critical length that prevents snap-back in the constitutive softening response, given by ${2G}_{I}E_{I}/\sigma_{I}$ [23], where *I* = *ft, fc, mt, mc*. This criterion was considered when determining the element size for modeling the composite plies. In the simulations, an element was removed from the mesh once the stiffness at all integration points had degraded to its maximum level, indicating complete failure.

**Table 3:** Composite material properties used in numerical analyses [24,25].

Elastic (MPa)					
$E_{11}=E_{22}$	$\vartheta_{12},\vartheta_{13},\vartheta_{23}$	$G_{12}$	$G_{13}$	$G_{23}$	
55,000	0.3	3363	3363	3363	
Strength (MPa)					
$X^{T}$	$X^{C}$	$Y^{T}$	$Y^{C}$	$S^{L}$	$S^{T}$
650	650	650	650	150	150
Damage (N/mm)					
*G_ft_*	*G_fc_*	*G_mt_*	*G_mc_*		
92	80	0.52	1.61		

### 3.2. Interply Damage

Cohesive surfaces were incorporated at the interfaces between composite layers to simulate their traction–separation response. Delamination at these interfaces was quantified using a specific parameter that represented the extent of damage. Within the finite element model, the quadratic nominal stress criterion was employed, which indicates that interfacial damage initiates once the normalized stress reaches a value of one [18]. The following expression, tailored for cohesive surfaces, represents this criterion:

(4) $${\left(\frac{\langle t_{1}\rangle }{t_{1}^{0}}\right)}^{2}+{\left(\frac{\langle t_{2}\rangle }{t_{2}^{0}}\right)}^{2}+{\left(\frac{\langle t_{3}\rangle }{t_{3}^{0}}\right)}^{2}=1.$$

Here, $t_{i}$, i=1,2,3 represent the normal and the first and second shear traction components at the cohesive surface, while $t_{i}^{0}$ denote the corresponding interfacial strengths marking the onset of separation for each mode: Mode 1 (normal), Mode 2 (first shear), and Mode 3 (second shear).

The progression of damage based on fracture energy was simulated using a mixed-mode damage formulation, incorporating a power-law fracture criterion to account for the combined effects of different loading modes.

(5) $${\left(\frac{G_{1}}{G_{1}^{0}}\right)}^{\eta }+{\left(\frac{G_{2}}{G_{2}^{0}}\right)}^{\eta }+{\left(\frac{G_{3}}{G_{3}^{0}}\right)}^{\eta }=1$$

In this expression, $G_{i}$ represents the instantaneous fracture energies corresponding to different failure modes, while $G_{i}^{0}$ denotes the critical fracture energy values necessary to initiate failure in Modes I, II, and III, respectively. The parameter *η* defines the material’s sensitivity to mode mixity (assumed to be 2.0 in this study).

The traction–separation behavior at the cohesive interface is characterized by a linear elastic response in the normal and two shear directions, denoted by stiffness values *K*_11_, *K*_22_, and *K*_33_, respectively. This linear response continues until the curve reaches its maximum, indicating the onset of damage. At this point, $t_{i}^{0}$ corresponds to the peak traction values at the initiation of separation. Beyond this stage, damage evolution begins, and the material stiffness begins to degrade. The progression of damage is quantified by a scalar variable, *CSDMG*, which ranges from 0 (undamaged) to 1 (fully damaged), and is expressed as follows:

(6) $$CSDMG=\frac{\delta_{m}^{f}(\delta_{m}^{max}-\delta_{m}^{0})}{\delta_{m}^{max}(\delta_{m}^{f}-\delta_{m}^{0})}$$

In this formulation, $\delta_{m}^{max}$ denotes the maximum effective displacement experienced during the process, while $\delta_{m}^{f}$ and $\delta_{m}^{0}$ correspond to the effective displacements at complete failure and at the onset of damage, respectively. The interactions between traction $t_{i}$, the damage variable *CSDMG*, stiffness $K_{ii}$, and displacement ($\delta_{i}$ in both the normal and shear directions are governed by the following relationships:

(7) $$t_{i}=(1-CSDMG)K_{ii}\delta_{i}$$

The effective displacement represents a combined measure of the normal and shear deformations at the cohesive interface, and is calculated using the following expression:

(8) $$\delta_{m}=\sqrt{{\langle \delta_{1}\rangle }^{2}+{{(\delta }_{2})}^{2}+{{(\delta }_{3})}^{2}}$$

The cohesive properties applied in the finite element model, along with their corresponding values, are summarized in Table 4.

In the simulations, the delamination factor (*DF*) can be calculated from the ratio of the delaminated area (*D*_A_) to the nominal hole area (*N*_A_), i.e., *D*_A_/*N*_A_ [14].

## 4. Result and Discussion

In this section, three key aspects were examined in sequence: the difference in damage characteristics between punching and drilling in CFRP laminates, the effect of ply count on drilling-induced delamination, and the influence of spindle speed on the delamination behavior of CFRP during drilling. Both experimental observations and numerical simulations were carried out to investigate these topics. Damage characteristics were assessed based on the distribution of the CSDMG obtained from simulations as well as the corresponding DF values calculated, while in the experiments, SEM was employed to evaluate the extent and nature of delamination.

### 4.1. Validation of the Numerical Model Developed

**Figure 5:**
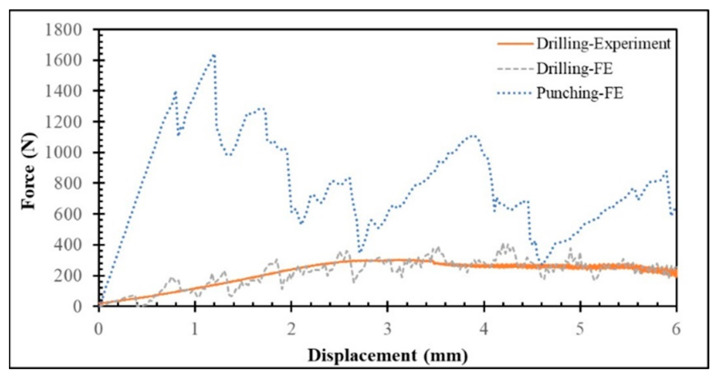
Force–displacement curves obtained from the punching and drilling (2500 rpm) of 4-ply CFRP plate.

This section presents the experimental and numerical evaluation of the force–displacement responses obtained during drilling of a 4-ply CFRP plate at 2500 rpm. As illustrated in Figure 5, a generally good agreement is observed between the experimental and FE simulation results, particularly in the initial loading and peak load regions. Both curves exhibit comparable stiffness in the elastic range up to approximately 2–3 mm displacement, and the FE model successfully captures the overall trend and maximum force observed experimentally. However, the numerical response shows more pronounced fluctuations and localized peaks. These oscillations primarily originate from numerical instabilities rather than physical material vibrations, typically caused by abrupt element deletion or stiffness degradation when progressive damage models activate sudden failure, leading to discontinuous load drops. Additional contributors include contact instabilities between the drill and workpiece—especially under hard or frictional contact formulations—and boundary reflections, both of which can produce artificial stress–wave interactions. Overall, the FE model reliably predicts the primary mechanical behavior and load-bearing capacity but could benefit from enhanced damping treatment and refined post-peak damage modeling to achieve a smoother softening response consistent with experimental observations. In experiments, the data acquisition system and transducers usually apply low-pass filtering, which removes high-frequency components, leading to smoother curves compared to raw numerical data.

To further examine the deformation behavior of the CFRP laminate, the force–displacement curves obtained from both punching and drilling were compared (Figure 5). The comparison clearly reveals distinct mechanical responses for the two processes. The punching response exhibits pronounced oscillations and abrupt force drops, corresponding to localized damage events such as fiber breakage, matrix cracking, and interlaminar delamination. These rapid load variations indicate unstable damage propagation and localized tearing as the punch penetrates successive plies. Such behavior is typical of the high-energy, impact-driven nature of punching, where multiple damage mechanisms—fiber shearing, resin cracking, and interface debonding—are activated almost simultaneously.

A closer analysis of the punching curve reveals a stepwise load evolution rather than a smooth monotonic increase. As the punch engages each ply, the load rises sharply until the local stress exceeds the ply’s failure strength, after which a sudden fracture and perforation occur, leading to a temporary force drop. The process repeats as the punch advances through subsequent plies, producing a characteristic fluctuation pattern in the force–displacement curve, notably around displacement values of 2.8 mm and 4.8 mm, consistent with the laminate’s ply thickness intervals. Each load peak corresponds to the onset of penetration into a new ply, while each subsequent drop reflects complete failure and transition into the interlaminar region with reduced resistance.

In contrast, the drilling response follows a smoother and more gradual trend, indicative of a stable, continuous material removal process dominated by chip formation and fiber–matrix abrasion. Unlike punching, where failure occurs abruptly, drilling removes material progressively, minimizing sudden stress redistributions and resulting in fewer load oscillations. As the punching process progresses and additional plies are fractured, the overall resistance decreases, leading to a gradual decline in load with increasing displacement. Once the final ply is perforated, the load drops sharply, signifying complete penetration. The observed punching behavior aligns well with the experimentally reported results of Ayten et al. [17].

Quantitatively, the punching process requires a significantly higher force (718.6 N) than drilling (206.9 N), which has implications for both tool wear and process efficiency. The higher forces involved in punching accelerate tool degradation and may limit its suitability for high-precision or repetitive operations. Nevertheless, its simplicity and potential cost-effectiveness make punching an attractive option for rapid hole formation in composite laminates when surface finish and delamination control are not primary concerns. Conversely, drilling, while operating under lower loads and offering superior tool life, demands longer processing times and more controlled operational parameters.

In this part of the study, the contact conditions during the drilling process were examined. According to the literature, the coefficient of friction between the tool and the workpiece typically ranges from 0.2 to 0.7 [26,27]. Therefore, the effect of varying the coefficient of friction within this range on the thrust force was investigated for cutting parameters of 2500 rpm and 12.5 mm/s. The corresponding results are summarized in Table 5. It was found that as the friction coefficient (*μ*) increased from 0.2 to 0.4 and subsequently to 0.7, the thrust force decreased from 293.5 N to 206.9 N and further to 128.1 N. Although some previous studies have indicated that the coefficient of friction has a negligible effect on thrust force, the present results revealed a decreasing trend with increasing μ. This behavior can be explained by the intensified shear stress at the tool–workpiece interface as μ increases, which accelerates the accumulation of shear energy and leads to faster element deletion in the contact region. Consequently, the effective load-bearing area beneath the drill reduces more rapidly, lowering the overall thrust force. The shorter time to failure at higher friction coefficients reflects the faster damage evolution driven by the elevated interfacial stresses. These shear stresses contribute to the progressive damage predicted by the Hashin failure theory, as described in Section 3.1. A similar trend was also reported by [27].

At high spindle speeds, however, thermal effects such as matrix softening and fiber–matrix interface degradation may additionally influence the drilling response. Elevated temperatures can locally reduce the matrix stiffness and interlaminar strength, facilitating easier material removal and potentially contributing to the observed reduction in thrust force at higher friction conditions. Although these thermal–mechanical interactions were not explicitly modeled in the present work, they merit detailed investigation in future studies to better capture the coupled thermal and damage mechanisms in high-speed drilling of CFRP composites.

### 4.2. Comparison of Damage Characteristics of CFRP Subjected to Punching and Drilling

In this section, the damage behavior of CFRP laminates subjected to punching and drilling is compared. Emphasis is placed on delamination patterns. This comparison aims to highlight the differences in failure mechanisms associated with each process and to provide insight into the suitability of these techniques for CFRP.

**Figure 6:**
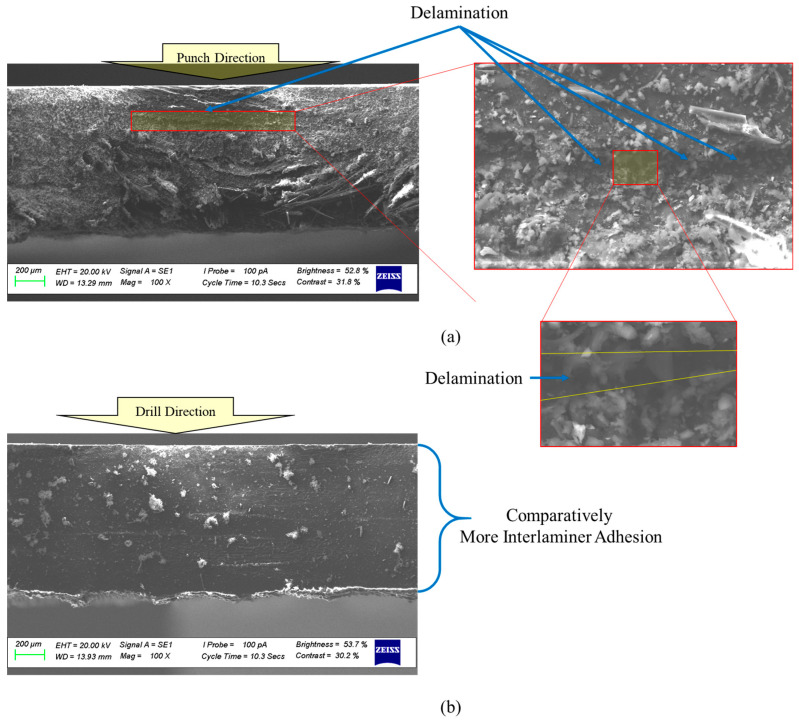
Comparison of SEM images of 4-ply CFRP surfaces machined by (**a**) punching and (**b**) drilling at 2500 rpm.

Figure 6 shows SEM images of CFRP surfaces with 4 plies after being processed by punching and drilling. In Figure 6a, the surface punched reveals significant delamination, which stems from the sudden, concentrated force applied during the punching process—often exceeding the interlaminar strength of the composite. On the other hand, Figure 6b shows that drilling, which removes material more gradually and applies cutting forces more evenly, results in much less delamination. This contrast highlights how punching tends to be more damaging than drilling, underlining the importance of using controlled cutting methods to better preserve the structural integrity of composite laminates.

**Figure 7:**
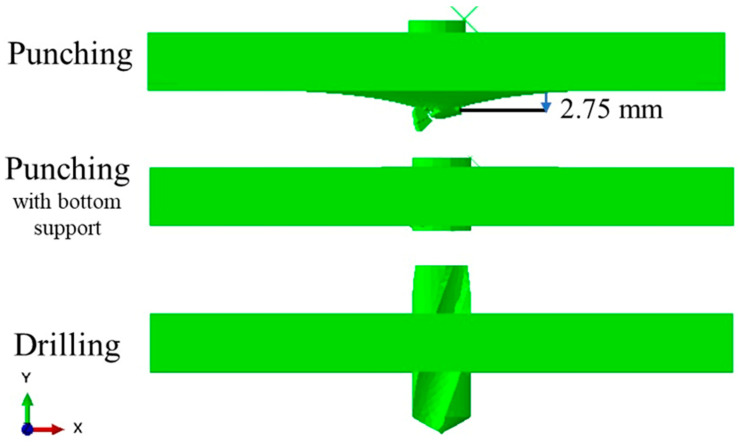
The deformed shape of CFRP plate with 4 plies subjected to punching and drilling.

FE simulations were performed to investigate how different hole-making strategies affect the structural response of a 4-ply CFRP laminate. Two conventional approaches—punching and drilling—were first compared to highlight their distinct deformation mechanisms (see Figure 7). In the punching case, the laminate exhibited a pronounced downward deflection of approximately 2.75 mm. This significant displacement occurs because punching delivers a sudden, concentrated force through the full thickness of the plate, forcing it to bend before the material is sheared. Such deformation can introduce additional stresses that compromise laminate integrity beyond the immediate cutting zone. By contrast, drilling generated an entirely different deformation profile. The rotating tool gradually removes material from the plate while distributing cutting forces along its edges. This progressive removal of material prevents the accumulation of high out-of-plane stresses, resulting in a much flatter plate during and after hole formation. This difference in global behavior suggests that drilling inherently offers a more controlled and stable process compared to the abrupt, forceful nature of punching. However, punching remains an attractive option in industry due to its speed and simplicity. This raises a critical question: can the detrimental effects of punching—particularly the large bending and interlaminar stresses—be mitigated without abandoning its efficiency advantage?

To explore this, an additional configuration was introduced: punching with bottom support. As seen in Figure 7 (middle), the presence of a rigid backing substantially alters the mechanics of deformation. The laminate remains almost flat during punching because the support constrains downward displacement, reducing bending-induced tensile stresses between layers. This simple modification demonstrates that the main weakness of the punching process—excessive out-of-plane deformation—can be significantly alleviated through fixture design, bridging the gap between high-speed hole creation and laminate integrity.

**Figure 8:**
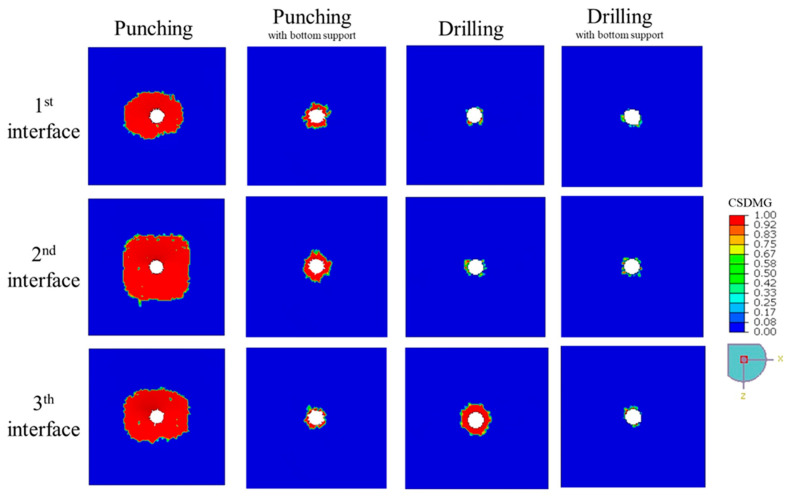
The degree of delamination, represented by the distribution of *CSDMG*, across different interface layers of a 4-ply CFRP plate (with and without bottom surface support) subjected to punching and drilling at 2500 rpm.

The impact of different hole-making configurations on delamination is vividly demonstrated in Figure 8, where the *CSDMG* plots illustrate the extent and pattern of damage across the 1st, 2nd, and 3rd interface layers of the 4-ply CFRP plates. This visual evidence is complemented by the quantitative results presented in Table 6, which reports the *DF* for each layer. Among all the methods, unsupported punching produced the most severe damage, with *DF* values escalating from 16.81 at the first interface to a peak of 31.09 at the second interface, and slightly decreasing to 22.97 at the third interface. This extensive delamination is primarily due to the pronounced global bending and interlaminar opening stresses that occur when no support is provided, causing the composite layers to peel apart and crack around the hole perimeter.

In contrast, drilling significantly confined the delamination to the immediate vicinity of the hole. The *DF* values for drilling were substantially lower, measured at 1.15 and 1.67 for the first and second interfaces, respectively, which indicates that the cutting mechanism of the drill bit imposes less severe interlaminar stresses. However, the third interface exhibited a higher *DF* of 3.25, likely due to the push-out forces generated as the drill exits the laminate. These localized forces can induce fiber pull-out or matrix cracking near the hole exit, which is a known challenge in composite drilling.

The addition of bottom support during punching demonstrated a remarkable improvement, reducing *DF* values to 2.29, 3.36, and 1.56 across the three interfaces. This reduction confirms that proper fixturing can mitigate global bending and distribute the applied force more evenly, thereby minimizing delamination. Similarly, drilling with backing plate yielded *DF* values of 1.09, 1.31, and 1.37, showing that even drilling benefits from additional support in maintaining the structural integrity of the laminate, particularly at the hole exit.

Overall, these findings highlight a key insight: while punching is inherently a more aggressive process, its negative impact on composite integrity is not inevitable. By integrating effective support strategies, punching can achieve delamination levels closer to those observed with drilling, while retaining its advantages of simplicity and speed. However, for applications where the highest laminate integrity is critical, particularly at the entry surface, drilling remains the preferred method due to its ability to maintain minimal delamination without requiring extensive fixture adjustments.

**Figure 9:**
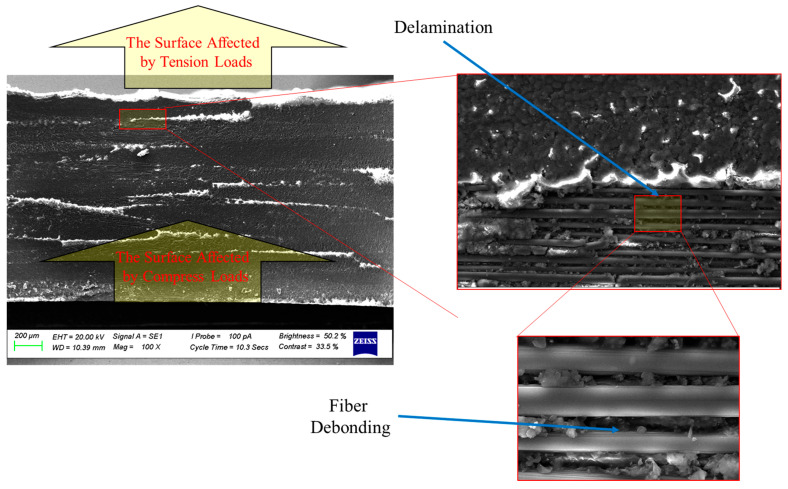
SEM images of 6-ply CFRP surfaces machined by drilling at 2500 rpm.

The experimental investigations in the current study reveal that delamination gradually spreads from the surface experiencing compression to the surface subjected to tension, as illustrated in Figure 9 showing SEM images of 6-ply CFRP surfaces machined by drilling at 2500 rpm. Notably, the amount of delamination is significantly greater on the tension-loaded surface. This phenomenon can be explained by the higher strain energy release rates associated with tensile loading, along with the reduced constraints in this state, which facilitate easier interlaminar separation compared to the compressed side.

### 4.3. Effect of Ply Count on Delamination Behavior

**Figure 10:**
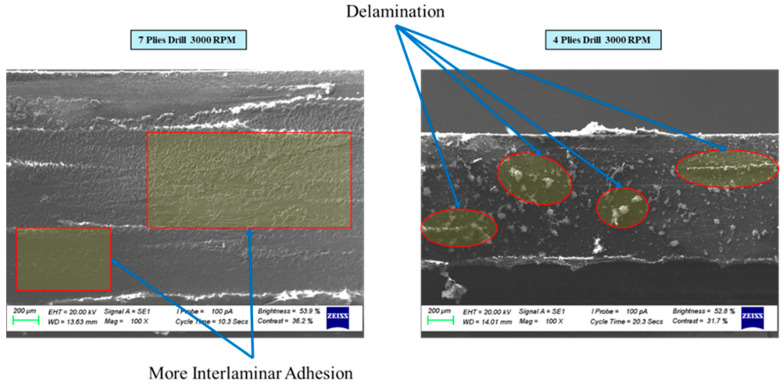
SEM images of the drilled surfaces of 4-ply and 7-ply CFRP plates subjected to drilling at 3000 rpm.

The influence of ply count on delamination in CFRP laminates during drilling was examined in this section. Figure 10 presents SEM images of 4-ply and 7-ply CFRP surfaces after drilling at a spindle speed of 3000 rpm. As shown, delamination is noticeably reduced in the 7-ply sample compared to the 4-ply one. This improvement in damage resistance can be explained by the added support and structural stability provided by the additional plies. Each extra interface helps maintain interlaminar adhesion more effectively during drilling, resulting in a less damaged surface.

**Figure 11:**
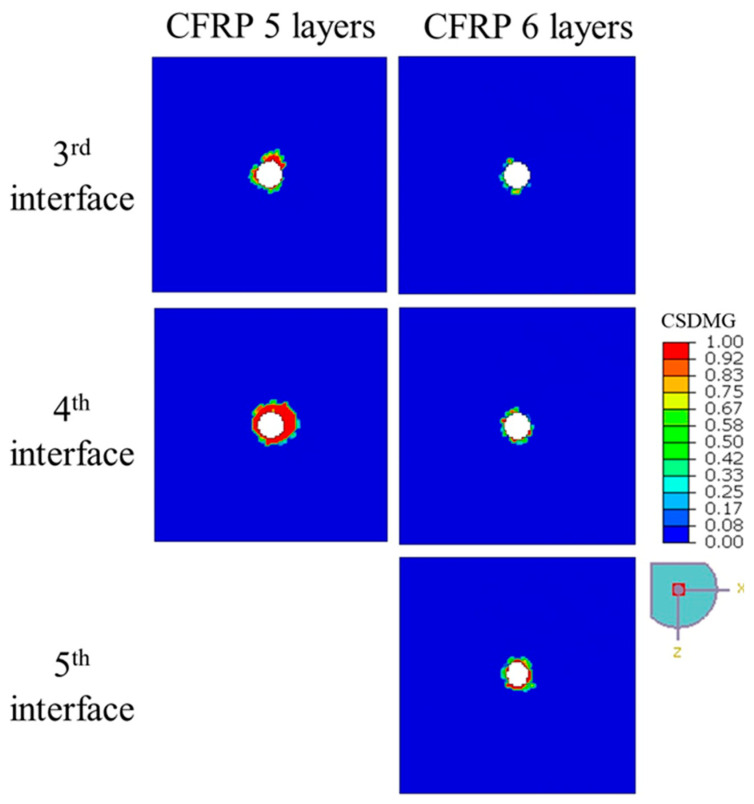
The extent of delamination—represented by the distribution of *CSDMG*—across various interface layers of 5- and 6-ply CFRP plates subjected to drilling at 2500 rpm.

In the simulations, the drilling performance of CFRP plates with 4, 5, and 6 plies was compared. Figure 8 and Figure 11, together with the data in Table 6, give a clear picture of how laminate thickness influences drilling-induced delamination. For the 4-ply laminate, the damage pattern changes significantly as we move deeper into the material. The first and second interfaces show only minimal delamination, with *DF* values of 1.15 and 1.67—barely more than a slight halo around the hole. However, at the third interface, things look very different: the *DF* jumps to 16.45, and the *CSDMG* plots reveal a large, concentrated damaged zone. This behavior makes sense because, as the drill approaches the exit side of a thin laminate, there is less material to resist the thrust force, and structural confinement weakens. As a result, the push-out effect dominates, driving severe interlaminar stresses that trigger rapid crack growth and delamination. In short, thinner laminates are far more vulnerable to damage at the exit plane during drilling.

The picture changes noticeably with thicker laminates. In the 5-ply laminate, the first two interfaces show virtually no measurable damage (hence no *DF* values), and even the deeper layers perform much better. At the third interface, the *DF* is just 1.54, and at the fourth interface, it is 3.36—numbers that are significantly lower than those seen in the 4-ply case. This suggests that having more plies stiffens the structure and helps distribute drilling forces more evenly through the thickness, reducing local stress peaks. The 6-ply laminate takes this improvement a step further. Here, delamination is barely present across all observed layers: *DF* values stay close to 1.0–1.5, and the damage maps show almost no significant failure zones. Essentially, the extra layers provide greater confinement and multiple “barriers” that slow down or even stop crack propagation.

These results point to a simple but important insight: thicker laminates handle drilling better. They absorb energy more effectively, reduce stress concentrations, and keep damage confined near the hole, instead of allowing it to spread into large delaminated regions. For industries where hole quality and structural integrity matter—such as aerospace and automotive—choosing a thicker laminate design, or at least accounting for ply count during drilling operations, can make a big difference in performance and durability.

**Figure 12:**
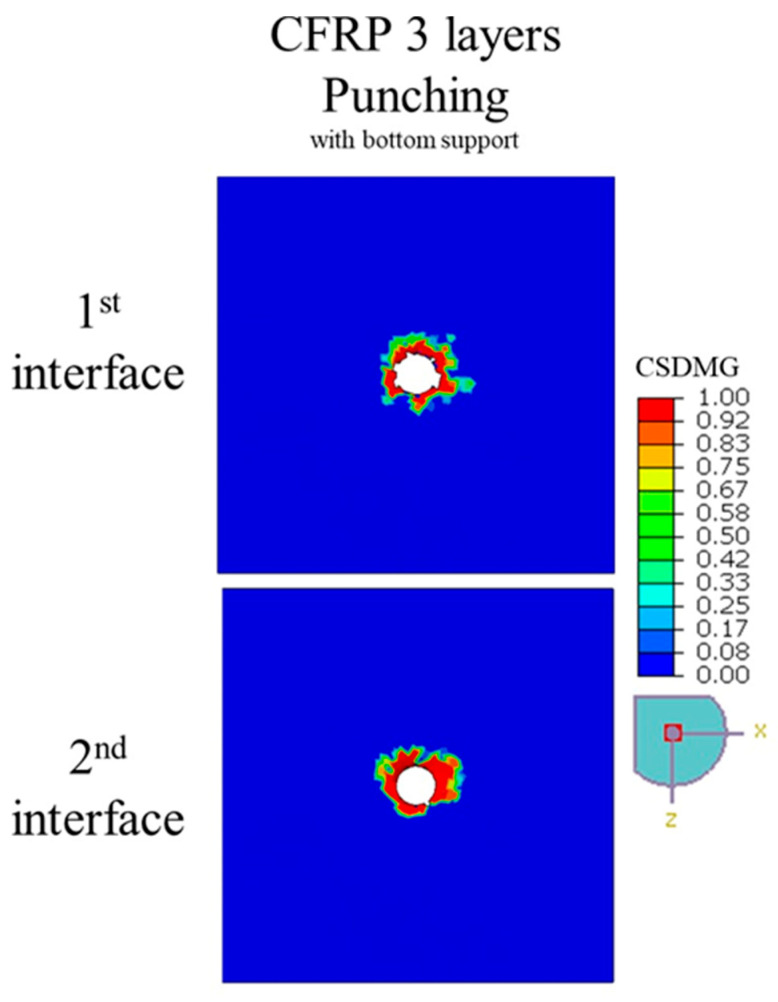
The delamination extent, as illustrated by the distribution of *CSDMG*, across various interface layers of 3-ply CFRP plates with bottom surface support during punching.

Figure 8 and Figure 12, along with Table 6, compare the delamination behavior of 3-ply and 4-ply CFRP laminates under punching with bottom surface support. The distribution of *CSDMG* across the interface layers indicates that the number of plies does not significantly influence the extent of delamination. While minor differences can be observed—for instance, the 4-ply laminate shows slightly higher delamination factors *DF*s of 2.29 and 3.36 at the first and second interfaces compared to 2.15 and 3.15 for the 3-ply laminate—these variations are not substantial enough to suggest a strong dependency on ply count. This can be attributed to the nature of the punching process, which is primarily an impact-driven event rather than a material removal operation, causing localized damage concentrated around the punch area irrespective of the total laminate thickness. A 3-ply laminate was chosen for comparison with the 4-ply laminate because the simulation of a 5-ply configuration could not be completed due to severe element distortions, resulting from the highly non-linear deformation during impact. This limitation arises because punching involves instantaneous force transfer and interlaminar shearing, which lead to excessive mesh distortion in thicker laminates during the numerical simulation. Overall, the results suggest that delamination propagation during punching is governed more by local stress concentration and interlaminar strength than by the total number of plies.

### 4.4. Influence of Drilling Speed and Feed Rate in Punching Processes

**Figure 13:**
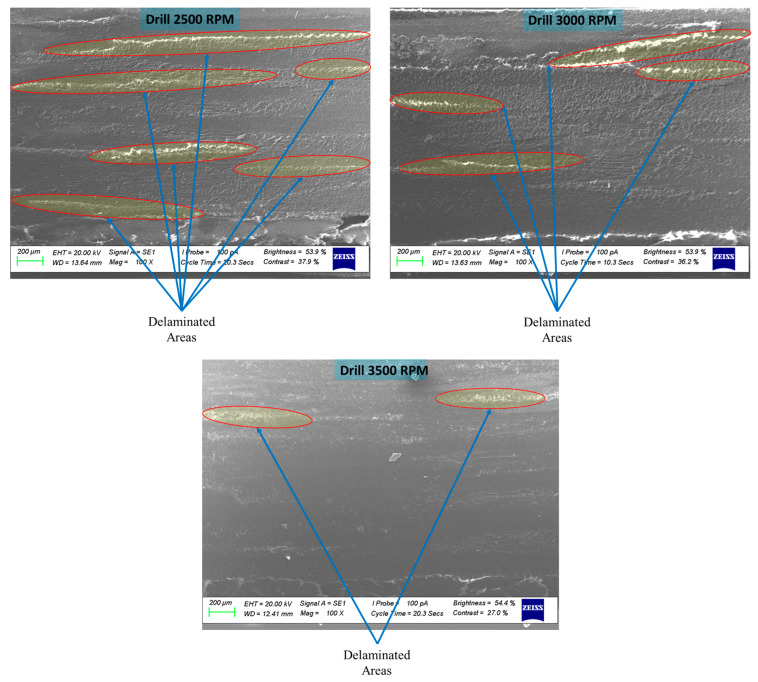
SEM images of the drilled surfaces of 7-ply CFRP plates subjected to drilling at 2500 rpm, 3000 rpm, and 3500 rpm.

This section examines how drilling speed influences delamination in 4-ply CFRP plates. Figure 13 shows microscopic images of surfaces drilled at 2500, 3000, and 3500 rpm, highlighting a clear decrease in delamination as the spindle speed increases. When the speed was raised from 2500 to 3500 rpm, the area of delamination around the holes was notably reduced. This improvement is primarily due to lower thrust forces and more efficient material removal at higher speeds, which help maintain better interlaminar adhesion and limit fiber debonding. These results align with previous findings [28], which also reported that higher drilling speeds mitigate delamination in composite laminates by reducing the mechanical stresses responsible for interlaminar failure.

**Figure 14:**
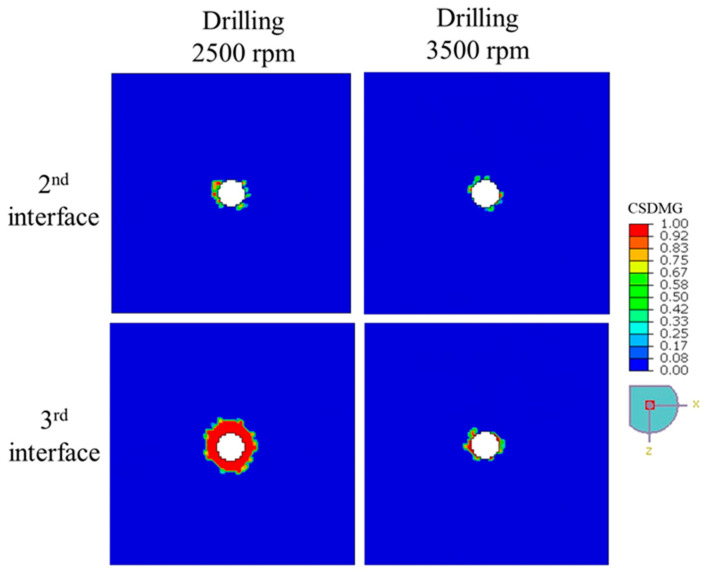
The extent of delamination—represented by the distribution of *CSDMG*—across various interface layers of a 4-ply CFRP plate subjected to drilling at 2500 rpm and 3500 rpm.

Figure 14 illustrates the distribution of delamination damage, quantified by *CSDMG*, across the 2nd and 3rd interface layers of the 4-ply CFRP plate subjected to drilling at rotational speeds of 2500 rpm and 3500 rpm. The results clearly show that increasing the drilling speed leads to a noticeable reduction in the extent of delamination, particularly at the more deeply embedded 3rd interface. At 2500 rpm, the delaminated region is significantly larger, with high *CSDMG* values concentrated around the hole, especially at the 3rd interface where red zones (*CSDMG* ≈ 1) dominate. In contrast, drilling at 3500 rpm results in a more localized and less severe damage pattern, indicating that higher spindle speeds may reduce the severity of interlaminar failure by limiting the mechanical energy transferred into delamination during the drilling process.

**Figure 15:**
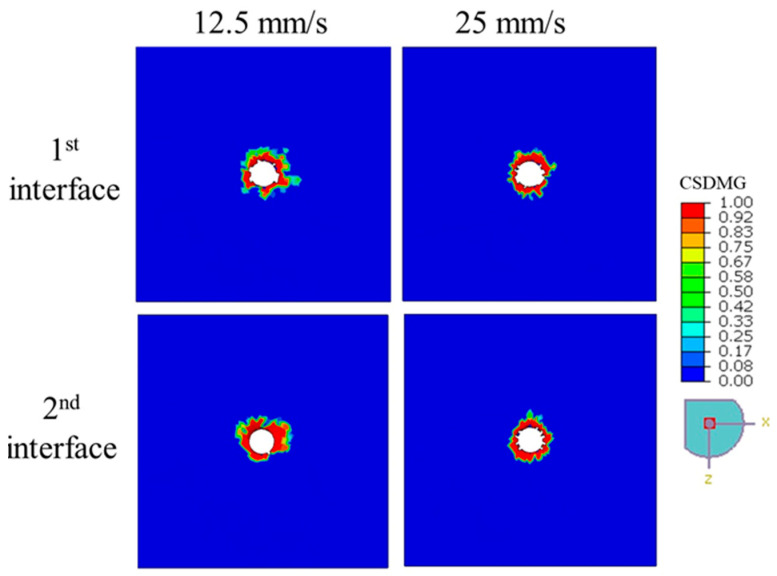
The degree of delamination—indicated by the *CSDMG* distribution—within the interface layers of a 3-ply CFRP plate punched at feed rates of 12.5 mm/s and 25 mm/s.

The extent of delamination in punched 3-ply CFRP laminates under different feed rates is depicted in Figure 15, which shows the distribution of the *CSDMG* values across the first and second interface layers. At both 12.5 mm/s and 25 mm/s feed rates, localized damage is observed around the tool contact region, with high *CSDMG* values (approaching 1.0) concentrated at the hole edges. Although slight variations in damage patterns are visible—particularly in the spread and symmetry of delaminated regions—there is no clear trend indicating a significant influence of feed rate on the severity of delamination. This observation is further supported by the data in Table 6, where the delamination factors at the first and second interfaces remain relatively close for both feed rates. For instance, the first interface shows a *DF* of 2.15 at 12.5 mm/s and 2.27 at 25 mm/s, while the second interface displays a decrease from 3.15 to 2.89, respectively. These small differences suggest that, within the examined range, feed rate does not substantially alter the delamination behavior during the punching process. Therefore, it can be inferred that the delamination resistance of CFRP under punching is relatively insensitive to moderate changes in feed rate, which may benefit process stability and part quality.

## 5. Conclusions

CFRP laminates are widely used in aerospace and high-performance applications but are prone to delamination during processes such as drilling and punching. This study examines how ply count and drilling speed affect delamination in 3- to 7-ply CFRP laminates using both experimental and numerical approaches. Tests were performed with 8 mm tools at various spindle speeds, and SEM was used to assess damage, while a 3D finite element model in ABAQUS/Explicit simulated delamination using Hashin and cohesive zone criteria. The following were concluded:

- SEM and FE results reveal that impact punching causes bending and widespread delamination due to concentrated loading, while drilling applies gentler, distributed forces that limit damage; using a rigid backing plate during punching reduces out-of-plane deflection and helps preserve laminate integrity.
- With proper backing plate, punching can achieve delamination levels comparable to drilling, offering a simpler and faster alternative for thinner CFRP plates; however, its higher forces increase tool wear and limit its feasibility for thicker plates, making drilling the preferred method when laminate integrity, tool life, and material thickness are critical.
- SEM observations and simulation data show that increasing the number of plies (from 4 to 7) significantly reduces delamination, particularly at the hole exit. Additional plies enhance structural stiffness and distribute drilling forces more evenly, mitigating push-out effects and interlaminar stresses.
- Increasing drilling speed significantly reduces delamination in CFRP laminates—particularly at deeper interfaces—by lowering thrust forces and mechanical stresses, while variations in feed rate during punching have minimal impact on delamination severity, indicating stable damage behavior across tested conditions.
- In future work, we plan to extend the comparison of the drilling and punching performance of composite plates to include a wider spectrum of laminate architectures (such as angle-ply layups), a broader range of laminate thicknesses and number of plies, to better reflect practical industrial applications. Furthermore, the influence of different tool geometries, feed rates, and environmental conditions (including cooling, lubrication, and temperature) will be systematically investigated to provide deeper insights into the process–material interactions and to enhance the generalizability and industrial relevance of the comparative findings.

## Figures and Tables

**Table 1:** Hashin 3D failure criteria [18,19].

Failure Mode	Condition	Failure Criteria
*ft*	$\sigma_{11}\geq 0$	${f_{1}=\left(\frac{\sigma_{11}}{X^{T}}\right)}^{2}+\alpha {\left(\frac{\sigma_{12}}{S^{L}}\right)}^{2}$
*fc*	$\sigma_{11}<0$	$f_{2}={\left(\frac{\sigma_{11}}{X^{C}}\right)}^{2}$
*mt*	$\sigma_{22}\geq 0$	$f_{3}={\left(\frac{\sigma_{22}}{Y^{T}}\right)}^{2}+{\left(\frac{\sigma_{12}}{S^{L}}\right)}^{2}$
*mc*	$\sigma_{22}<0$	$f_{4}={\left(\frac{\sigma_{22}}{{2S}^{T}}\right)}^{2}+\left[{\left(\frac{Y^{C}}{{2S}^{T}}\right)}^{2}-1\right]\frac{\sigma_{22}}{Y^{C}}+{\left(\frac{\sigma_{12}}{S^{L}}\right)}^{2}$

**Table 2:** Equivalent displacement and stress for each of the four damage modes [18].

Failure Mode	Condition	$\sigma_{I,eq}$	$\delta_{I,eq}$
*ft*	$\sigma_{11}\geq 0$	$\frac{L_{c}(\langle \sigma_{11}\rangle \langle \varepsilon_{11}\rangle +{\alpha \sigma }_{12}\varepsilon_{12})}{\delta_{ft,eq}}$	$L_{c}\sqrt{{\langle \varepsilon_{11}\rangle }^{2}+{\alpha \varepsilon }_{12}^{2}}$
*fc*	$\sigma_{11}<0$	$\frac{L_{c}\langle {-\sigma }_{11}\rangle \langle -\varepsilon_{11}\rangle }{\delta_{fc,eq}}$	$L_{c}\langle -\varepsilon_{11}\rangle$
*mt*	$\sigma_{22}\geq 0$	$\frac{L_{c}(\langle \sigma_{22}\rangle \langle \varepsilon_{22}\rangle +\sigma_{12}\varepsilon_{12})}{\delta_{mt,eq}}$	$L_{c}\sqrt{{\langle \varepsilon_{22}\rangle }^{2}+\varepsilon_{12}^{2}}$
*mc*	$\sigma_{22}<0$	$\frac{L_{c}(\langle {-\sigma }_{22}\rangle \langle \varepsilon_{22}\rangle +\sigma_{12}\varepsilon_{12})}{\delta_{mc,eq}}$	$L_{c}\sqrt{{\langle {-\varepsilon }_{22}\rangle }^{2}+\varepsilon_{12}^{2}}$

$L_{c}$: the characteristic length, computed as a square root of the integration point area. $\varepsilon_{ij}$: various strain values. $\langle t\rangle$: the Macaulay bracket operator defined as (t +$\;\left|t\right|)/2.$

**Table 4:** Interface properties of glass fiber reinforced composite used in numerical analyses [11,14].

$K_{11}$, $K_{22}$, $K_{33}$ (MPa/mm)	$t_{1}^{0}$ (MPa)	$t_{2}^{0}=t_{3}^{0}$ (MPa)	$G_{1}^{0}\;$ (N/mm)	$G_{2}^{0}$, $G_{3}^{0}$ (N/mm)
1 × 10^3^	60	110	0.33	1.209

**Table 5:** Influence of friction coefficient on the thrust force from drilling (2500 rpm) of 4-ply CFRP plate.

*μ*	Avg. Thrust Force (N)
0.2	293.5
0.4	206.9
0.7	128.1

**Table 6:** Numerically obtained *DF* values at different interface layers of CFRP plate for different configurations.

	Input			Delamination Factor (*DF*)				
				Interface Layers				
Type of Process	Number of Layers	Feed Rate (mm/s)	Drilling Speed (rpm)	1st	2nd	3rd	4th	5th
Punching	4	12.5	-	16.81	31.09	22.97	-	-
Punching with bottom support	4	12.5	-	2.29	3.36	1.56	-	-
Drilling	4	12.5	2500	1.15	1.67	3.25	-	-
Drilling with bottom support	4	12.5	2500	1.09	1.31	1.37	-	-
Drilling	5	12.5	2500	-	-	1.54	3.36	-
Drilling	6	12.5	2500	-	-	1.09	1.33	1.45
Punching with bottom support	3	12.5	-	2.15	3.15	-	-	-
Drilling	4	12.5	3500	-	1.41	10.38	-	-
Punching with bottom support	3	25.0	-	2.27	2.89	-	-	-

## Data Availability

The raw data supporting the conclusions of this article will be made available by the authors on request.
